# Improved Bond Strength of Cyanoacrylate Adhesives Through Nanostructured Chromium Adhesion Layers

**DOI:** 10.1186/s11671-016-1629-9

**Published:** 2016-09-20

**Authors:** Kyle Gobble, Amelia Stark, Stephen P. Stagon

**Affiliations:** Mechanical Engineering, University of North Florida, Jacksonville, FL 32224 USA

**Keywords:** Physical vapor deposition, Nanorods, Adhesion, Polyolefin, Cyanoacrylate

## Abstract

The performance of many consumer products suffers due to weak and inconsistent bonds formed to low surface energy polymer materials, such as polyolefin-based high-density polyethylene (HDPE), with adhesives, such as cyanoacrylate. In this letter, we present an industrially relevant means of increasing bond shear strength and consistency through vacuum metallization of chromium thin films and nanorods, using HDPE as a prototype material and cyanoacrylate as a prototype adhesive. For the as received HDPE surfaces, unmodified bond shear strength is shown to be only 0.20 MPa with a standard deviation of 14 %. When Cr metallization layers are added onto the HDPE at thicknesses of 50 nm or less, nanorod-structured coatings outperform continuous films and have a maximum bond shear strength of 0.96 MPa with a standard deviation of 7 %. When the metallization layer is greater than 50 nm thick, continuous films demonstrate greater performance than nanorod coatings and have a maximum shear strength of 1.03 MPa with a standard deviation of 6 %. Further, when the combination of surface roughening with P400 grit sandpaper and metallization is used, 100-nm-thick nanorod coatings show a tenfold increase in shear strength over the baseline, reaching a maximum of 2.03 MPa with a standard deviation of only 3 %. The substantial increase in shear strength through metallization, and the combination of roughening with metallization, may have wide-reaching implications in consumer products which utilize low surface energy plastics.

## Background

Joining together two surfaces using a polymeric adhesive, such as cyanoacrylate, has been commonplace for decades, but the resulting bonds still suffer from major performance issues [1]. For example, bonds made with low surface energy polyolefin-based materials, such as high-density polyethylene (HDPE), suffer from insufficient bond strength with a large standard deviation [2, 3]. In many engineering applications, like in some medical devices, adhesive bonding is the only method that is acceptable due to the high cost of alternative methods and additional constraints like dissimilarity of the materials to be bonded [4, 5]. Often, the combination of adhesives that bond to a wide range of materials, like cyanoacrylate, and low surface energy polymeric materials, like HDPE, are necessitated by the constraints of the design. Unfortunately, the bonds formed between cyanoacrylate and HDPE are mechanically weak with large sample to sample strength variation, causing the performance of the end product to suffer drastically [2, 3]. The low bond strength between these materials derives from the absence of polar groups on the polymer surface and the weak chemical interaction between the polymer and adhesive [6, 7].

To increase the overall strength of adhesive joints, either the interface bond strength, being the intrinsic strength of the physical bonds between the polymer and the adhesive, may be increased or the surfaces may be modified to create microscale mechanical interlocking of the polymer and the adhesive [7–12]. Beginning with the simpler and cheaper technique, in mechanical roughening, an abrasive with microscale features is used to increase the area of the adhesive-polymer interface [8, 9]. While the local interface interaction between the surfaces is not changed, the global connection between the two surfaces is increased by creating torturous features and long-range interpenetration of the adhesive and polymer. Although mechanical roughening is widely used, the results are less than ideal due to a high sensitivity to the morphology of the microscale features and a loss of part tolerance, which may be of importance to the final product [8]. A method of altering the intrinsic strength of the chemical bonds is modification of the polymer surface through either wet chemical treatment, plasma treatment, or metallization. Due to the low reactivity of the polyolefin surface, both chemical and plasma modification of HDPE remain difficult [7, 10–12]. Alternatively, in metallization, a thin layer of metal, often chromium (Cr), titanium (Ti), or tantalum (Ta), is added to the surface through either electrochemical or vacuum deposition and forms a strong physical bond with the surface [13–15]. When the adhesive is applied, a strong bond forms with the metal layer. Metallization is applicable for parts in which tolerances are very important or mechanical modification is unacceptable, or it may be used in combination with mechanical roughening to further increase joint strength.

Two main methods exist for metallization of polymeric parts, being vacuum metallization through physical vapor deposition (PVD) and electroless plating. Electroless plating, while simple, is being phased out in many industries due to highly toxic intermediates, such as strong acids [16, 17]. PVD metallization presents a more sustainable alternative and preferable results due to the intrinsic purity of the vacuum process. Vacuum metallization of polymeric parts also becomes cost competitive with electrochemical methods through economies of scale [18].

The sensitivity of joint strength to the thickness and morphology of the metallization layer is not well known, especially in the case of metallization layers with nanostructured morphologies, like nanorods. In the case of a continuous film, stress may develop in the film during deposition, due to the mismatch between the film and the polymeric under-layer, and may cause long-range cracking and delamination [19]. Often, failure of the joint occurs through the delamination of the metal film from the surface in continuous large regions [20]. To improve the strength and performance of the joints, stress must be mitigated and a means of mitigating long-range delamination must be realized.

In this letter, using an inexpensive vacuum-deposited Cr adhesion layer, HDPE substrates, a cyanoacrylate adhesive, and lap shear tests, we design a nanostructured adhesion layer out of Cr nanorods to mitigate the effects of film stress and improve joint shear strength. These materials are chosen as they are exemplary prototype materials for industrial applications due to their widespread use. Further, we investigate the sensitivity of joint shear strength to film thickness, adhesion layer morphology, and the presence of surface roughness. To the authors’ knowledge, this is the first time that nanorods have been used to mitigate film stress and promote the bonding of adhesives to low surface energy polymers.

## Methods

Before presenting the results, we briefly discuss the methods used in the metallization and shear strength testing. Substrates for bonding are rectangular strips and are 10-cm long and 2.5-cm wide. The substrates to be coated are white HDPE (McMaster Carr Co. part number 8671K68) and have a thickness of 0.3 cm. The opposing substrates are 304 stainless steel with mirror finish (McMaster Carr Co. part number 9785K217) and have a thickness of 0.15 cm. The stainless steel substrates are abraded by hand with P400 grit sandpaper (McMaster Carr Co. part number 47025A44) with ten passes to the bonding surface in the same direction and are then cleaned sequentially with acetone and isopropanol. The same sanding protocol is also used for the portion of the HDPE substrates that are sanded prior to metallization. Immediately before metallization, all the HDPE substrates are sequentially cleaned with acetone and isopropanol.

The prepared HDPE substrates are then metallized using high-vacuum PVD through thermal evaporation. The vacuum chamber is a stainless steel tank of approximately 40 cm in diameter and 50 cm in height. The source to sample distance is approximately 30 cm. Source material, 99.995 % Cr (Kurt J Lesker Co.), is evaporated from tungsten coil sources. For deposition of continuous films, the substrates are placed perpendicular to the normal of the source. For the deposition of nanorods, the samples are placed into glancing angle configuration, such that the normal of their surfaces are oriented at 87° relative to the source normal [21]. The chamber is pumped down with a turbomolecular pump to a base pressure of 1 × 10^−4^ Pa, and the working pressure during deposition is ~1 × 10^−3^ Pa. Cr is deposited at a rate of 0.1 nm/s to thicknesses varying from 25 to 200 nm. The deposition rate and thickness are monitored via a quartz crystal microbalance, which is located perpendicular to the vapor flux.

Scanning electron microscopy (SEM) is performed soon after nanorod and film growth using a FEI Nova Nano microscope located in the Nanoscale Research Facility at the University of Florida. The microscope is operated under high vacuum, using immersion mode with a through the lens detector, at a working distance of 5 mm, an accelerating voltage of 10 kV, and a spot size of 2.0.

Adhesive bonding of the samples is performed in ambient using a lap shear configuration with 2.5 cm of overlap, following ASTM-D3165 standards. One droplet of the cyanoacrylate adhesive (Loctite 4161 Instant Adhesive, Henkel Co.), measured at 65 mm^3^, is placed onto each of the HDPE surfaces, and then the sanded steel surfaces are brought into contact. Next, a load of 25 N is applied to the overlapped region and maintained for 48 h. After 48 h, the load is removed and the samples are tested for shear strength using an Instron 3369 equipped with a 2530 low-profile 50-KN load cell. Force is measured at one data point per second under a constant displacement rate of 2.5 cm/m. Following failure, the bonded area of each sample is measured by taking an optical image, manually creating a perimeter around the wetted area, and measuring the area using built-in functions of the open source software package ImageJ (https://imagej.nih.gov/ij/).

## Results and Discussion

**Fig. 1:**
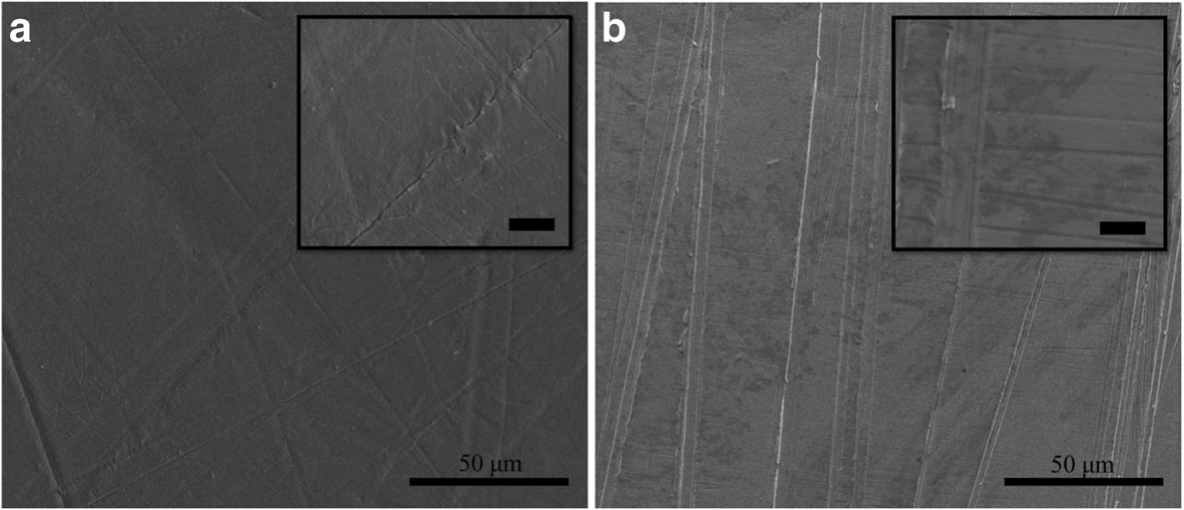
SEM images of bonding surfaces. SEM images of the substrates used for lap shear bonds with **a** the surface of an unmodified HDPE substrate, with a high-magnification inset where the *scale bar* is 5 μm, and **b** a prepared surface of 304 stainless steel, with a high-magnification inset where the *scale bar* is 5 μm. The HDPE surface has been prepared with 5 nm of gold deposited for SEM imaging

The first results presented are the unmodified surfaces, which are used for bonding, as shown in the SEM images of Fig. 1. The as received HDPE surface, Fig. 1a, exhibits micron- and nanometer-scale features from fabrication and industrial handling and is representative of an industrially available surface. The stainless steel surface, which has been prepared with P400 grit sandpaper, exhibits numerous micron-scale channels but few nanometer-scale features, as shown in Fig. 1b. We note that all adhesive joints failed through delamination or tearing of the HDPE surfaces—adhesion to the stainless steel was maintained in all tests.

**Fig. 2:**
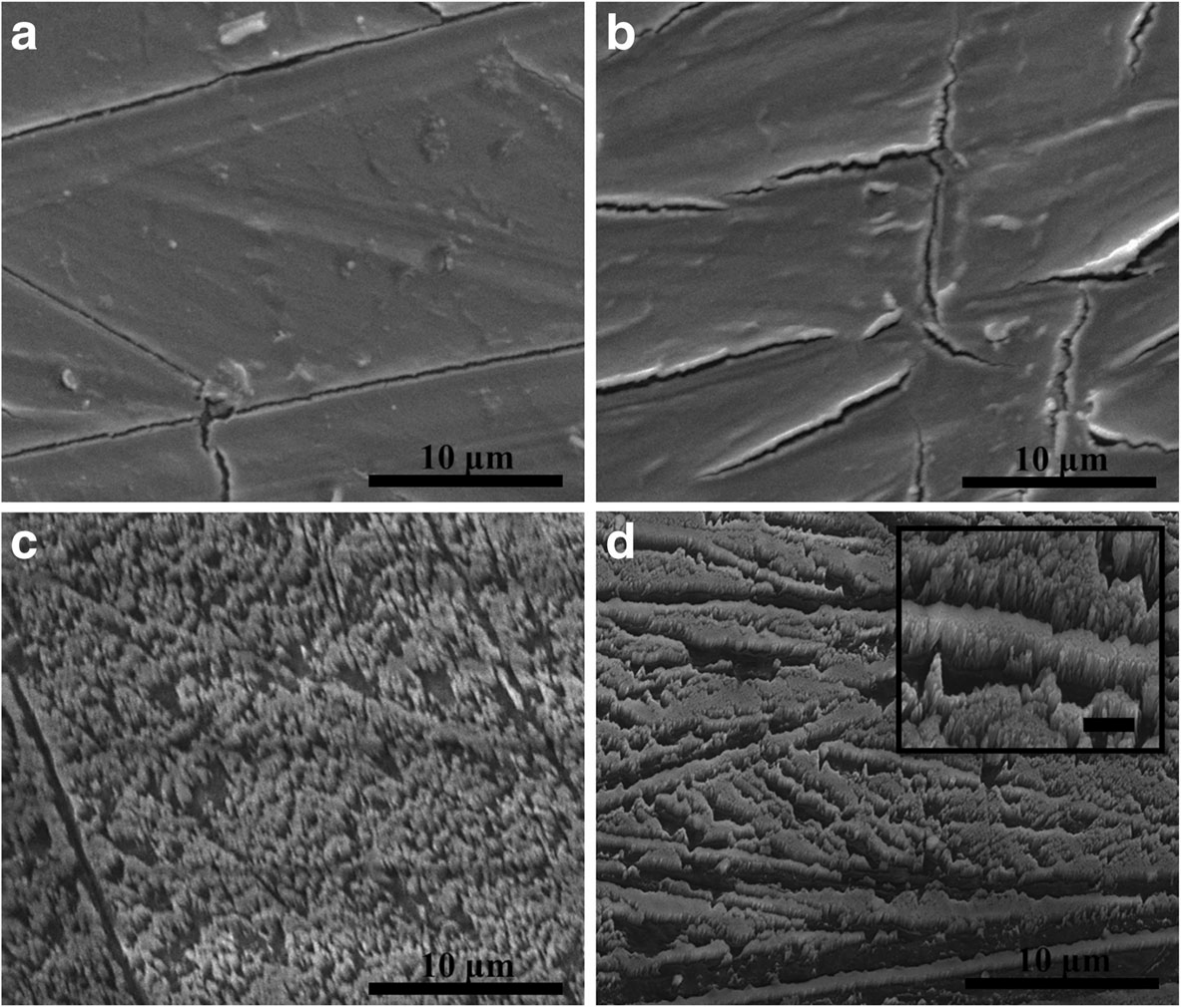
SEM images of nanorod- and film-coated surfaces. SEM images of Cr films deposited onto HDPE substrates to thicknesses of **a** 50 nm and **b** 200 nm and Cr nanorods deposited onto HDPE substrates to nominal thicknesses of **c** 50 nm and **d** 200 nm. **d** The *inset* is an SEM image at a higher magnification to highlight the morphology of the coalesced Cr nanorods, where the *scale bar* is 1 μm

To demonstrate the sensitivity of the joint shear strength to film thickness and morphology, Cr-continuous films and nanorods are deposited at thicknesses ranging from 25 to 200 nm onto the HDPE substrates, as shown in Fig. 2. When 25 nm is deposited, not shown here, the films are smooth and conformal to the substrate. When continuous films are deposited to thicknesses of 50 nm and greater, micron-scale cracking develops. As the films are grown thicker, the width of the cracks increases and the spacing between cracks decreases. When 50 nm of Cr is deposited as a film, the films remain conformal to the substrate and cracks are ~20 μm apart, Fig. 2a. When the thickness of the films is increased to 200 nm, the crack spacing decreases to ~5 μm and areas along the crack edges become irregular and wrinkled, Fig. 2b. The crack spacing for the 100-nm continuous films, not show here, is ~10 μm and has minimal wrinkling. In direct contrast to the films, when nanorods are deposited, they reside on the high areas of the substrate, due to the glancing angle nature of the deposition, and have more patchy coverage. In direct comparison to Fig. 2b, nanorods deposited to nominally 50 nm in length, Fig. 2c, are sparse and individual. As the nominal lengths of the nanorods are increased to 200 nm, Fig. 2d, the nanorods become clumped and resemble lines on the substrate. The inset of Fig. 2d, a higher magnification SEM image, shows the coalescence of the Cr nanorods.

**Fig. 3:**
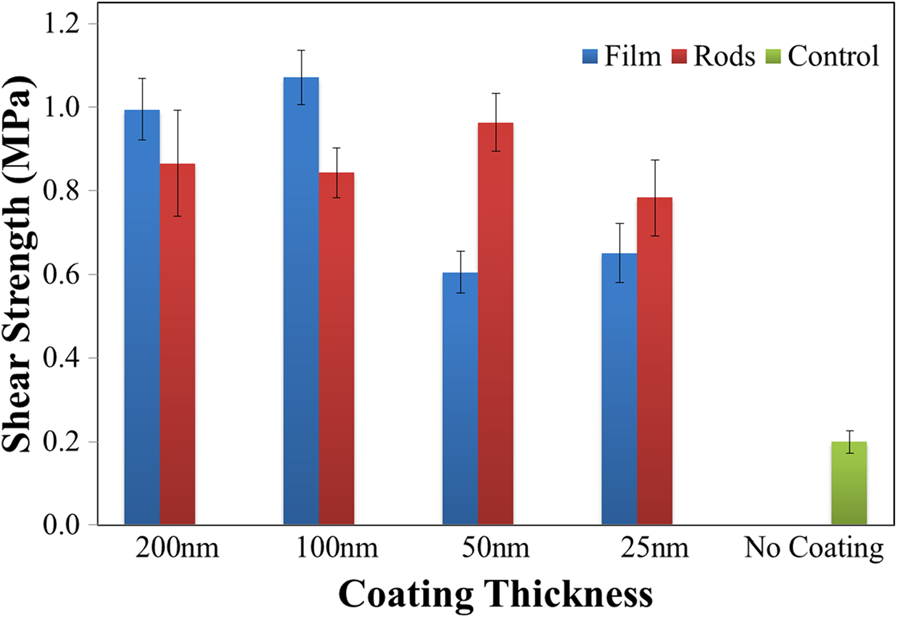
Graph of shear strength under different coating conditions. Shear strength of the joints created at the different conditions, including a baseline of no modification. Standard deviation of the strength is reported as the *scale bars*

Putting the surface modifications to the test, we next present the shear strength of the adhesive joints, Fig. 3. Here, rather than presenting the force required to fail the standard 2.5 × 2.5 cm joint, we present shear strength, which is the force divided by the bonded area, to eliminate differences in the wettability of the surfaces by the glue. In technological applications, the amount of glue and method of applying the glue may be slightly modified to assure total coverage of the joint, which is not done here for consistency. The baseline shear strength is extremely low, at only 0.20 MPa with a standard deviation of 0.03 MPa, or 14 %. When the thickness of the metallization layer is 50 nm or less, the rods have higher strengths than the continuous films, while both morphologies increase the strength of the bond by threefold or greater. Under this thickness condition, the domains of the film case are continuous and much larger than the domains of the nanorods, which are nearly entirely separate. When the thickness of the metallization layer is increased to 100 nm or greater, the films’ strength surpasses that of the nanorods. In this domain, there is substantially more coverage of the substrate by metal in the continuous film case than the nanorod case, while both cases are predominantly coalesced and continuous. The strength of the nanorod films reaches a global maximum at 50 nm, before coalescence begins, with a strength of 0.96 MPa and a standard deviation of 0.07 MPa, or 7 %. The strength of the films reaches a global maximum at 100 nm with a strength of 1.03 MPa and a standard deviation of 0.06 MPa, or 6 %. At 100 nm, the cracks are closely spaced but wrinkling of the crack edges is not evident.

As a final test, the HDPE surface is sanded with P400 grit sandpaper and then coated with 100 nm of Cr, as both a continuous film and rod, to determine if the combination of roughening and metallization is advantageous. When a film is used, the shear strength of the bond increases to 1.72 MPa with a standard deviation of 0.08 MPa, or 4 %. When rods are used, the shear strength increases to 2.03 MPa with a standard deviation of 0.06 MPa, or 3 %, which is the strongest bond achieved with the smallest standard deviation percentage in this study.

In passing, we also discuss the standard deviation of bond strength of the respective modifications, as this has significant impact on the engineering of consumer products, and the biocompatibility of this prototype bond. When the surfaces are not modified, the standard deviation is 14 %. When a continuous film of 100 nm is used on a flat HDPE surface and the maximum metallization strength is only reached, the standard deviation of the joint strength decreases to only 6 %. When 50-nm nanorods are used on flat HDPE and the maximum nanorod strength is reached, the standard deviation is only 7 %. When roughness is added to the HDPE substrate, the standard deviations decrease to only 4 and 3 % for the continuous film and nanorod cases, respectively. Switching to the discussion of biocompatibility, Cr is used as a prototype material in this work as it is of low cost and is thus applicable to a wide range of technologies. For applications that require biocompatibility, the use of Cr may not be advantageous, and it may be replaced by a more biocompatible refractory material, such as Ti.

## Conclusions

In this work, we present a means of increasing the shear strength of cyanoacrylate and HDPE joints through vacuum metallization of Cr-continuous films and nanorods. When the HDPE surfaces are not modified, the bond shear strength is only 0.20 MPa, with a standard deviation of 14 %. When the surfaces are metallized with Cr, the strength of the joints is dependent on both layer thickness and morphology. When the metallization layer is 50 nm or less, nanorods outperform films, reaching a maximum shear strength of 0.96 MPa with a standard deviation of 7 %. When the layer thickness is great than 50 nm, continuous films outperform nanorods, reaching a maximum shear strength of 1.03 MPa and a standard deviation of 6 % at a thickness of 100 nm. When both sanding and metallization are used in combination, the greatest shear strength is reached, with a maximum of 2.03 MPa and a standard deviation of 3 % for the case of 100-nm nanorods. For the case of engineered parts with close tolerances, vacuum metallization with both nanorods and continuous films offers a dramatic increase in shear strength and decrease in standard deviation, with nanorods performing best at thicknesses below 50 nm and films performing best at thicknesses of 100 nm and greater. For overall strength, where dimensional tolerances allow, the combination of sanding and metallizing with nanorods offers the greatest shear strength, with a tenfold increase in shear strength and an 11 % decrease in standard deviation from baseline.

## References

[1] Coover HW, Dreifus DW, O’connor JT (1990) Cyanoacrylate adhesives. Handbook of adhesives. Springer US. pp 463-477)

[2] Gutowski WS, Wu DY, Li S (1993). Surface silanization of polyethylene for enhanced adhesion. J Adhes.

[3] Wu DY, Gutowski WS, Li S, Griesser HJ (1995). Ammonia plasma treatment of polyolefins for adhesive bonding with a cyanoacrylate adhesive. J Adhes Sci Technol.

[4] Valdastri P, Harada K, Menciassi A, Beccai L, Stefanini C, Fujie M, Dario P (2006). Integration of a miniaturized triaxial force sensor in a minimally invasive surgical tool. IEEE Trans Biomed Eng.

[5] Valdastri P, Houston K, Menciassi A, Dario P, Sieber A, Yanagihara M, Fujie M (2007). Miniaturized cutting tool with triaxial force sensing capabilities for minimally invasive surgery. J Med Devices.

[6] Friedrich J (2014). Tailoring of interface/interphase to promote metal‐polymer adhesion. Adhesion in Microelectronics.

[7] Liu JC (1992) Loctite Corporation, assignee. Primer for bonding low surface energy plastics with cyanoacrylate adhesives and bonding method employing same. United States patent US 5,079,098.

[8] Uehara K, Sakurai M (2002). Bonding strength of adhesives and surface roughness of joined parts. J Mater Process Technol.

[9] Cardoso PE, Braga RR, Carrilho MR (1998). Evaluation of micro-tensile, shear and tensile tests determining the bond strength of three adhesive systems. Dent Mater.

[10] Okamoto Y, Klemarczyk PT (1993). Primers for bonding polyolefin substrates with alkyl cyanoacrylate adhesive. J Adhes.

[11] Woods JG, Fréchet JM (2004) Loctite Corporation, assignee. Multi-amine compound primers for bonding of polyolefins with cyanoacrylate adhesives. United States patent US 6,673,192.

[12] Mandolfino C, Lertora E, Gambaro C, Bruno M (2014). Improving adhesion performance of polyethylene surfaces by cold plasma treatment. Meccanica.

[13] Balloni R, Keung JK, Mount III EM (1989) Mobil Oil Corporation, assignee. Process for manufacturing a metallized polyolefin film and resulting film. United States patent US 4,888,237.

[14] Mallory W, Migliorini R, Parr L (2004) Exxonmobil Oil Corporation, assignee. Multilayer metallized polyolefin film. United States patent US 6,723,431.

[15] Vassiliadi E, Tarantili PA (2007). Characterization of metallized biaxially oriented polypropylene film. J Appl Polym Sci.

[16] Tengsuwan S, Ohshima M (2012). Electroless nickel plating on polypropylene via hydrophilic modification and supercritical carbon dioxide Pd-complex infusion. J Supercrit Fluids.

[17] Tengsuwan S, Ohshima M (2014). Environmentally benign electroless nickel plating using supercritical carbon-dioxide on hydrophilically modified acrylonitrile–butadiene–styrene. Appl Surf Sci.

[18] De Bruyn K, Van Stappen M, De Deurwaerder H, Rouxhet L, Celis JP (2003). Study of pretreatment methods for vacuum metallization of plastics. Surf Coat Technol.

[19] Marshall DB, Evans AG (1984). Measurement of adherence of residually stressed thin films by indentation. I. Mechanics of interface delamination. J Appl Phys.

[20] Gioia G, Ortiz M (1997). Delamination of compressed thin films. Adv Appl Mech.

[21] Niu X, Stagon SP, Huang H, Baldwin JK, Misra A (2013). Smallest metallic nanorods using physical vapor deposition. Phys Rev Lett.

